# ST-SCSR: identifying spatial domains in spatial transcriptomics data via structure correlation and self-representation

**DOI:** 10.1093/bib/bbae437

**Published:** 2024-09-04

**Authors:** Min Zhang, Wensheng Zhang, Xiaoke Ma

**Affiliations:** School of Computer Science and Technology, Xidian University, No. 2 South Taibai Road, 710071 Xi’an Shaanxi, China; Key Laboratory of Smart Human-Computer Interaction and Wearable Technology of Shaanxi Province, Xidian University, No. 2 South Taibai Road, 710071 Xi’an Shaanxi, China; School of Computer Science and Cyber Engineering, GuangZhou University, No. 230 Wai Huan Xi Road,Guangzhou Higher Education Mega Center, 510006 Guangzhou Guangdong, China; School of Computer Science and Technology, Xidian University, No. 2 South Taibai Road, 710071 Xi’an Shaanxi, China; Key Laboratory of Smart Human-Computer Interaction and Wearable Technology of Shaanxi Province, Xidian University, No. 2 South Taibai Road, 710071 Xi’an Shaanxi, China

**Keywords:** spatial transcriptomics, spatial domain, sparse representation, joint learning, matrix factorization

## Abstract

Recent advances in spatial transcriptomics (ST) enable measurements of transcriptome within intact biological tissues by preserving spatial information, offering biologists unprecedented opportunities to comprehensively understand tissue micro-environment, where spatial domains are basic units of tissues. Although great efforts are devoted to this issue, they still have many shortcomings, such as ignoring local information and relations of spatial domains, requiring alternatives to solve these problems. Here, a novel algorithm for spatial domain identification in Spatial Transcriptomics data with Structure Correlation and Self-Representation (ST-SCSR), which integrates local information, global information, and similarity of spatial domains. Specifically, ST-SCSR utilzes matrix tri-factorization to simultaneously decompose expression profiles and spatial network of spots, where expressional and spatial features of spots are fused via the shared factor matrix that interpreted as similarity of spatial domains. Furthermore, ST-SCSR learns affinity graph of spots by manipulating expressional and spatial features, where local preservation and sparse constraints are employed, thereby enhancing the quality of graph. The experimental results demonstrate that ST-SCSR not only outperforms state-of-the-art algorithms in terms of accuracy, but also identifies many potential interesting patterns.

## Introduction

Exploring complex pathological structures is crucial for understanding biology and pathological mechanisms, which is also an important basis for revealing potential causes and pathophysiological changes [1, 2]. In multicellular organisms, cells are usually organized into different groups (known as cell types) [3], which can be effectively tracked with the single-cell RNA-sequencing (scRNA-seq) techniques by dissociating cells from tissues to measure the transcriptional levels of genes [4]. However, scRNA-seq lose spatial coordination of cells, failing to fully characterize micro-environments of tissue. Fortunately, recent advances in spatial transcriptomics (ST) enable the simultaneous measurement of expression profile and spatial information of cells, offering a new perspective to model the complicated structure of tissues [5].

On the basis of principles of approaches, current ST technologies can be roughly divided into imaging- and next generation sequencing (NGS)-based methods [6], where the former ones include fluorescence in situ hybridization [7] and its variant multiplexed error-robust fluorescence in situ hybridization [8, 9]. However, imaging-based techniques are critical for their limitation for genome coverage. In other words, these approaches only measure transcriptional levels of some genes, rather than the whole genome transcription. To address the aforementioned problem, NGS-based technologies, including Slide-seq [10], Stereo-seq [11], and 10 $\times $ Visium [12], employ spatial barcoding to cover whole genome transcription, facilitating the exploitation of structure and functions of tissues.

Spatial domains refer to continuous regions in tissue with the same or similar expression profiles, and identifying spatial domains in ST is the fundamental step for elucidating genomic diversity and cellular mechanisms [13]. And, available approaches are classified into two categories, i.e. biological experiments and computational methods, where the biological experimental approaches utilize biological manners to validate existence of spatial domains, and the latter ones employ machine learning to predict spatial domains from data. The advantage of biological experimental methods is reliable, while it is criticized for the low efficiency and time-consuming. As an alternative, the computational method typically utilizes machine learning tools to model and identify spatial domains in ST data, which is equivalent to the typical clustering problem [14, 15].

Clustering aims to assign objects into groups such that objects within the same group are highly similar, and dissimilar across various clusters in terms of features of objects. However, identifying spatial domain from ST data is non-trivial because spatial information and transcriptional information of spots are difficult to balance. On the basis of strategies for balancing these two issues, current algorithms for spatial domain identification are mainly categorized into two categories, including non-spatial and spatial clustering methods, where the first category simply ignores spatial information and the latter one integrates spatial and expression information. The non-spatial clustering methods, such as K-means [16], Louvain [17], SCANPY [18], and DRjCC [19], identifying spatial domains by only use the expression profile, ignoring the additional spatial information, result in an undesirable performance. In other words, these algorithms sacrifice accuracy for simplicity, i.e. they simply treat ST data as scRNA-seq data.

To overcome this limitation of non-spatial-based methods, spatial-based algorithms integrate spatial, expression, and/or additional information to fully characterize and identify spatial domains. And, current algorithms adopts various strategies to integrate these heterogeneous information. For example, Giotto [20] adopts the Hidden Markov Random Field model to model the coherence of spots in terms of gene expression, while stLearn [21] utilizes morphological features from histology images and spatial information to smooth expression profiles. BayesSpace [22] and SpatialPCA [23] employ probabilistic model to infer relations among spots, where proximal spots are more likely to receive higher probabilities, thereby forcing these proximal spots with the similar expression to form a spatial domain. These algorithms dramatically improve accuracy of identifying spatial domains, indicating that spatial information and additional information are crucial for spatial domains.

However, these algorithms only exploit pairwise relations among spots, rather than indirected relations among them. To address this limitation, network-based algorithms construct spot networks, and then learn features of spots by exploiting topological structure of networks, where indirected relations among spots are explore, thereby enhancing discriminative of features. For example, SpaGCN [13] integrates gene expression, spatial location, and histological images with the graph convolutional network (GCN), while CCST [24] uses graph neural networks (GNN) for spatial domain identification. STAGATE [25] utilizes a graphic attention auto-encoder framework to integrate spatial information and gene expression profiles, and SEDR [26] uses a deep auto-encoder network and a graphic auto-encoder to embed spatial information. In addition, GraphST [27] first combines GNN and contrastive learning to enhance representation of features of spots, and DeepST [28] effectively enhances data compatibility by combining cell expressional, spatial, and morphological information with GNN. These algorithms further improve performance of approaches, demonstrate that network model is promising for characterizing and identifying spatial domains. However, these algorithms fail to fully address heterogeneity of ST data, therefore decreasing compatibility of features. To address this problem, we propose a multi-layer network model (MNMST) [29], which independently constructs dual graphs for spatial and expression information, and then transforms spatial domain identification into multi-layer network clustering problem.

Although great efforts are committed to spatial domain identification from ST data, there still have many unsolved problems. First, current graph-based algorithms are devoted to learn features of objects to fully represent topological structure of spot network(s), ignoring local information of spots that is complementary to global information. Second, available algorithms solely focus on learning discriminative features of spots, which implicitly assume that the down-stream tasks are independent of feature learning. Actually, in our previous studies [19, 30], we show that down-stream tasks serve as prior information to guide feature learning, thereby enhancing quality of features. Third, current algorithms project spatial and transcriptional information into a shared subspace to obtain the low-dimensional features of spots, ignoring the meta-structure of spots, such as relations among spatial domains, which cannot fully characterize intrinsic properties of ST data.

**Figure 1:**
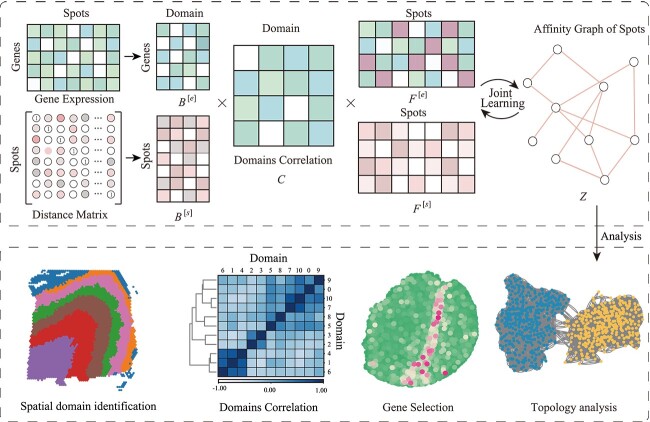
Overview of ST-SCSR. It consists of four major components, including network construction, feature learning, affinity graph learning, and down-stream analysis, where the first procedure constructs spatial network of spots, feature learning obtains compatible features with matrix tri-factorization, affinity graph learning captures relations among spots by integrating spatial and expression information, and down-stream analysis is executed on the affinity graph to identify spatial patterns.

To address these challenges, a novel algorithm, called ST-SCSR, is developed for identifying spatial domains from ST data, which integrates local information, global information and relations among spatial domain. As shown in Fig. 1, four procedures, such as network construction, feature learning, affinity graph learning and down-stream analysis, constitute ST-SCSR, where the first procedure construct spatial network of spots, feature learning procedure obtains compatible features of spots with matrix tri-factorization, and affinity graph learning extracts topological structure of spots by manipulating spatial and expressional features. Specifically, matrix tri-factorization strategy fuse expression profile and spatial network via the shared factor matrix $C$ that is interpreted as the similarity of meta-structure of spots, thereby enhancing compatibility of features of spots. Furthermore, the local, global and meta-structure information are integrated, providing a more comprehensive way to characterize spatial domain. Finally, experimental results demonstrate that the proposed algorithm is more accurate to identify spatial domains in ST data, indicating that down-stream tasks are promising for characterizing and analyzing ST data.

In all, the major contribution of this study can be summarized as

– ST-SCSR proposes a matrix tri-factorization strategy to integrate expression profile and spatial information of spots, where fusion of heterogeneous data is executed at the meta-structure level, rather than on the subspaces. Thus, it provides a new perspective for analyzing ST data.
– ST-SCSR integrates local information, global information and down-stream task to learn features of spots, thereby improving the quality and compatibility of features of spots.
– Compared to state-of-the-art baselines, ST-SCSR not only outperforms current algorithms, but also identifies more potential interesting patterns, which may provide biologists with clues.

## Materials and methods

### Data pre-processing

The spots out of tissue regions are deleted for all datasets, and gene expressed in less than 10 spots are also removed, and the top 3000 highly variable genes are selected for down-stream analysis. The normalization of gene expression is performed by using SCANPY [18] with respect to library size. The expression profiles of spots are augmented by using neighbor information through BANKSY [31]. Principal component analysis is adopted ro reduce the dimensionality of expression profiles.

### Mathematical model for ST-SCSR

As shown in Fig. 1, ST-SCSR is composed of three major components, i.e. network construction, feature learning, and affinity graph learning, where the first procedure construct spatial network for spots to capture spatial proximity of spots, the second one obtains features of spots with matrix tri-factorization, and the last one automatically learns affinity graph of spots that is the foundation for down-stream analysis, such spatial domain identification, topological analysis, etc.

On the network construction issue, ST-SCSR aims to establish spatial network for spots, where the proximity of spots in spatial space is preserved. In details, given gene expression profile $X \in \mathbb{R}^{m \times n}$ for $m$ genes and $n$ spots, ST-SCSR constructs the spatial network of spots $G=(V,E)$ with K-Nearest Neighborhood (KNN) by setting parameter K=5, where weight on edge $(v_{i},v_{j})\in E$ connecting the $i$th and $j$th spot is $w_{ij}=(\|\mathbf{x}_{.i}-\mathbf{x}_{.j}\|)^{-1}$ ($\mathbf{x}_{.i}$ is the $i$th column of $X$, i.e. expression profile of the $i$th spot), and $\|X\|^{2}$ denotes $l_{2}$-norm of matrix $X$. In other words, edge weights in adjacent matrix $W$ of $G$ are inversely proportional to distance of spots, where only proximal spots receive heavy weights.

On the feature learning issue, ST-SCSR performs matrix tri-factor decomposition on gene expression $X$ and adjacent matrix $W$ of spatial network $G$ of spots. The traditional nonnegative matrix factorization (NMI) [32] independently decomposes $X$ and $W$ into product of two low-rank nonnegative matrices, i.e. 


(1) \begin{align*}& \begin{cases} \|X-B^{[e]}F^{[e]}\|^{2},& B^{[e]}\geq 0, F^{[e]}\geq 0,\\ \|W- B^{[s]}F^{[s]}\|^{2},& B^{[s]}\geq 0, F^{[s]}\geq 0, \end{cases}\end{align*}

where $B^{[e]}(B^{[s]})$ and $F^{[e]}(F^{[s]})$ are the basis and coefficient matrix for expression $X$ (adjacent matrix $W$), respectively.

However, Equation(1) has several typical limitations. First, it ignores the relations among spatial and expression information, resulting in incompatible features of spots. Second, relations among spatial domains are also neglected, failing to characterize meta-structure of spatial domains. Third, local information of spots is also ignored, decreasing discriminative of features of spots. To address these issues, ST-SCSR adopts matrix tri-factorization to simultaneously decompose expression profile $X$ and adjacent matrix $W$ of graph $G$ as 


(2) \begin{eqnarray*} & \min & \|X-B^{[e]}CF^{[e]}\|^{2} + \|W-B^{[s]}CF^{[s]}\|^{2} \\ & s.t. & B^{[e]} \geq 0, B^{[s]} \geq 0, F^{[e]} \geq 0, F^{[s]} \geq 0, C = C^{^{\prime}},\nonumber \end{eqnarray*}

where $C$ is the common factor matrix, $C^{^{\prime}}$ is the transpose of matrix $C$, and constraint $C = C^{^{\prime}}$ ensures the symmetry of matrix $C$. In this case, spatial and expression information are simultaneously integrated with joint matrix factorization, where the common factor matrix $C$ bridges the spatial and expression features of spots. Furthermore, the common factor matrix $C$ can be interpreted as similarity of spatial domains. In the experimental results, we demonstrate that $C$ precisely characterizes relations among spatial domains.

Although Equation(2) successfully addresses the first two concerning, it still ignores the local information of spots. Therefore, we also expect expressional feature of spots preserve topological structure of $G$, i.e. if a pair of spots are proximal in $G$, they are also similar in the feature space. Fortunately, local preservation [33] can be formulated as trace optimization as 


(3) \begin{align*}& Tr(F^{[e]}L_{W}(F^{[e]})^{^{\prime}}),\end{align*}

where $L_{W}=D-W$ ($D$ is the degree diagonal matrix of $G$) is the Laplacian matrix of $G$, and $Tr(A)$ is trace of matrix $A$, i.e. $Tr(A)=\sum _{i}a_{ii}$.

On the affinity graph learning issue, ST-SCSR automatically learns a graph of spots from expressional and spatial features of spots, where down-stream analysis can directly be executed. Self-representation learning [34] is employed to learn affinity graph $Z$, which is formulated as 


(4) \begin{eqnarray*} & \min & \|F^{[e]} -F^{[e]} Z\|^{2} +\|F^{[s]} -F^{[s]} Z\|^{2} \\ & s.t. & F^{[e]} \geq 0, F^{[s]} \geq 0, Z=Z^{^{\prime}},\nonumber \end{eqnarray*}

where constraint $Z=Z^{^{\prime}}$ guarantees undirected affinity graph. Equation(4) ensures that affinity graph integrates spatial and expressional features of spots, providing a comprehensive way to characterize spatial domains. Furthermore, to improve interpretability of affinity graph, we impose sparse constraint on $Z$ with $l_{1}$-norm as 


(5) \begin{align*}& \| Z \|_{1},\end{align*}

which is sum of absolute values of elements in matrix $Z$.

By combining Equations(2, 3, 4, 5), the objective function of ST-SCSR is formulated as 


(6) \begin{eqnarray*} &\min & \underbrace{\|X-B^{[e]}CF^{[e]}\|^{2} +\|W-B^{[s]}CF^{[s]}\|^{2}}_{matrix \quad tri-factorization} \nonumber \\ &+& \underbrace{\alpha Tr(F^{[e]}L_{W}(F^{[e]})^{^{\prime}})}_{local \quad preservation} \\ & +& \underbrace{\beta (\|F^{[e]} -F^{[e]} Z\|^{2} +\|F^{[s]} -F^{[s]} Z\|^{2})+ \gamma \| Z \|_{1}}_{affinity \quad graph \quad learning} \nonumber\\ &s.t. & B^{[e]} \geq 0, B^{[s]} \geq 0, F^{[e]} \geq 0, F^{[s]} \geq 0, C = C^{^{\prime}},\nonumber \\ && C = C^{^{\prime}}, Z = Z^{^{\prime}},\nonumber \end{eqnarray*}

where parameter $\alpha $, $\beta $ and $\gamma $ control importance of each item. Equation(6) is optimized using the alternating direction multiplier method (ADMM) [35], which is detailed supplementary materials.

### Clustering and visualization

ST-SCSR identifies spatial domains from the learned affinity graph $Z$ with the Leiden algorithm [36]. If the number of clusters is unknown, it determines the number of spatial domains with instability of matrix factorization [37]. The uniform manifold approximation and projection (UMAP) technique [38] is adopted for visualization of spatial domains.

### Differentially expressed genes and functional enrichment analysis

Differential expression analysis is performed for each spatial domain identified by ST-SCSR, where genes are differentially expressed if they express at least 80% spots of given domain, log fold change is $\geq $ 1.5, and FDR $\leq $ 0.05 (Wilcoxon rank-sum test for significance) [18]. To obtain functions of DEGs, gene enrichment analysis is employed by using clusterProfiler [39], where significance is obtained with hypergeometric test (adjust FDR $\leq $ 0.05).

### Baselines and criteria

The most popular algorithms for spatial domain identification are selected as baselines, including SCANPY [18], Giotto [20], stLearn [40], SEDR [26], BayesSpace [22], SpaGCN [13], and STAGETE [25], PROST [41], BANKSY[31], DeepST [28], and MNMST [29]. SCANPY, SEDR, BayesSpace, SpaGCN, STAGATE, Giotto, and stLearn are selected because these methods are graph-based approaches. BANKSY, DeepST, PROST, and MNMST are selected because these methods are currently competitive algorithms. For the sake of fairness, all these values of parameters take the best values suggested by the original studies.

To quantify performance of various algorithms, the adjusted Rand Index (ARI) [42] and normalized mutual information (NMI) [32] are selected as measurements.

## Results

### Overview of the ST-SCSR workflow

The ultimate goal of ST-SCSR is to effectively integrate spatial information and expression profiles of spots by smoothing meta-structure of spots, and construct an affinity graph of spots for down-stream analysis.

As shown in Fig. 1, ST-SCSR fulfills the goal with four procedures, such as network construction, feature learning, affinity graph learning and down-stream analysis, where the first one transforms spatial information of spots into a network (Material and method section). To improve compatibility of features, ST-SCSR employs matrix tri-factorization to integrate expression profiles and spatial network of spots, where fusion of heterogeneous information is performed at the meta-structure of spots, i.e. similarity of spatial domains. In this case, down-stream tasks can be implicitly incorporated into feature learning to further improve discriminative of features because the shared factor matrix $C$ can be interpreted as similarity of spatial domains. To balance the local and global information of spots, ST-SCSR utilizes $l_{1}$-norm and Laplacian trace constraint to ensure the sparsity and local topological preservation of features, providing a better way to characterize structure of spatial domains. Finally, ST-SCSR obtains an affinity graph of spots with self-representation learning by manipulating spatial and expressional features of spots, serving as the foundation for down-stream analysis.

Compared to current graph-based algorithms for spatial domain identification, such as MNMST [29], SpaGCN [13], the proposed algorithm has two typical advantages. First, current algorithms project all information into a common subspace to obtain compatible features of spots, where the relations between spatial and expressional information are implicitly addressed. However, ST-SCSR explicitly addresses relations of these information at the meta-structure of spots, facilitating the down-stream analysis. Second, current algorithms are sensitive to ST data generated from various platforms because they fail to balance of spatial and expression information, whereas ST-SCSR avoids this dilemma by fusing heterogeneous information with the shared factor matrix $C$, thereby improving accuracy and robustness of algorithms.

### Parameter analysis demonstrate ST-SCSR is stable

ST-SCSR has four parameters, where $k$ is the number of features, and $\alpha $, $\beta $, and $\gamma $ control importance of local preservation, affinity graph learning and sparsity constraint, respectively.

How parameter $k$ effects performance of ST-SCSR is investigated on the dorsal prefrontal cortex (DLPFC) dataset [43], where the ground truth spatial domains are known. Thus, we check how ARI of ST-SCSR changes as the number of features increases from 10 to 100 (Supplementary Fig. S1A), where performance of ST-SCSR first increases from 10 to 50, and then decreases if $k$ is larger than 50. If parameter $k$ is small, features of spots fail to fully characterize structure of spatial domains in ST data because of insufficient dimensions of features. Performance of ST-SCSR decreases when the number of features is large because redundancy of features of spots are very likely to trigger feature confliction, therefore resulting in an desirable performance. ST-SCSR reaches the best performance at $k$=50.

How these parameters effect performance of ST-SCSR on the DLPFC dataset is also studied with pairwise grid search (Supplementary Fig. S1B–D). It is easy to conclude as parameter $\alpha \in $[1, 20], $\beta \in $[0.1, 10], and $\gamma \in $[50, 80], ST-SCSR achieves an excellent performance. There are some good reasons to explain this tendency. When parameter $\alpha $ is too small, contribution of local information is subtle, thereby decreasing ARI of ST-SCSR. If value of parameter $\alpha $ is too large, objective function is dominated by local information, thereby reducing quality of features. Analogously, small value of parameter $\beta $ results in a low quality of graph of spots, whereas large one breaks balance of features of features and graph of spots. When the parameter $\gamma $ is small, the contribution of sparsity constraint is limited, resulting in undesirable performance. These results also demonstrate that ST-SCSR is stable since the ranges of parameters are sufficient large. Notice that we performs parameter effect on various datasets, and the tendency is similar. To remove redundancy, we only present results on DLPFC dataset. In all these experiments, we set $\alpha $=2, $\beta $=1, and $\gamma $=50.

On the efficiency issue, we independently execute each algorithm for each slide of DLPFC data, where distributions of running time and space of various algorithms are summarized in Supplementary Fig. S2. Giotto and BayesSpace are slower than stLearn, ST-SCSR and MNMST, and SCANPY, SpaGCN and DeepST are faster than others. Specifically, running time of ST-SCSR is 3.81 $\pm $ 1.54 minutes, while that is 0.07 $\pm $ 0.004 (SCANPY), 10.63 $\pm $ 2.87 (Giotto), 6.30 $\pm $ 2.16 (BayesSpace), 0.56 $\pm $ 0.07 (SEDR), 3.57 $\pm $ 0.41 (stLearn), 1.37 $\pm $ 0.33 (SpaGCN), and 0.14 $\pm $ 0.02 (STAGATE), respectively (Supplementary Fig. S2A). And, space complexity of Gitto, stLearn and DeepST much higher than others (Supplementary Fig. S2A). In details, space of ST-SCSR is 0.98 $\pm $ 0.35 Gigabytes, while that is 1.48 $\pm $ 0.16 (SCANPY), 5.51 $\pm $ 0.42 (Giotto), 1.20 $\pm $ 0.09 (BayesSpace), 1.90 $\pm $ 0.11 (SEDR), 8.55 $\pm $ 1.49 (stLearn), 1.69 $\pm $ 0.08 (SpaGCN), and 2.05 $\pm $ 0.05 (STAGATE), respectively. These results show that ST-SCSR reaches a good balance between efficiency and effectiveness.

### ST-SCSR precisely identifies spatial domains in various ST datasets for normal brain tissues generated with the 10$\times $ Visium platform

We first evaluate the performance of various algorithms on with including human DLPFC [43], which is generated with 10$\times $ Visium platform. It contains 12 slices sequenced from 3 human brains, where each slice is annotated as six cortical layers (first Layer to sixth Layer) and the White Matter Layer based on known markers and morphological features, which is selected as benchmark for algorithms. Eleven up-to-date algorithms, such as SCANPY, Giotto, stLearn, SEDR, BayesSpace, SpaGCN, DeepST, MNMST, BANKSY, STAGATE, and PROST are selected as baselines to fully validate the performance of the proposed algorithm (Material and Methods section).

**Figure 2:**
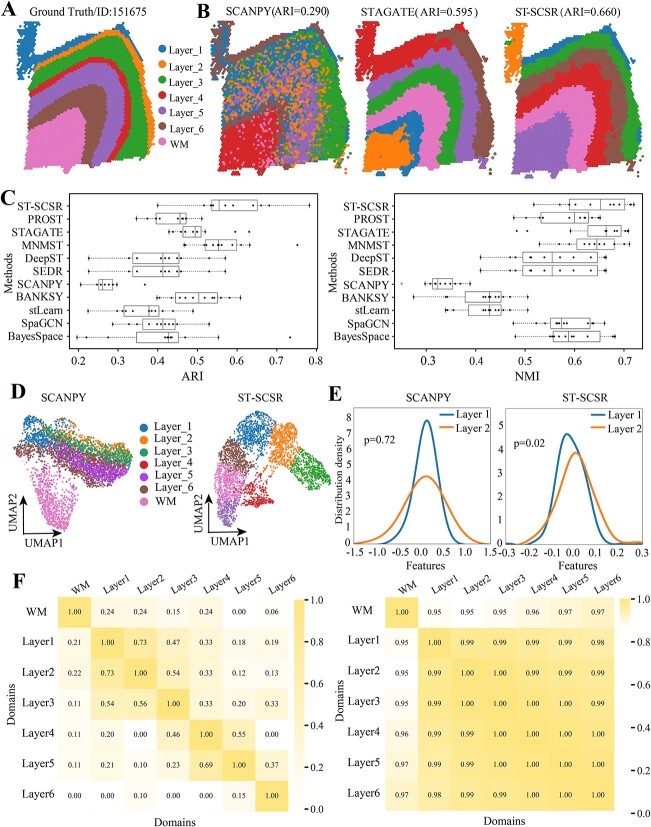
Performance of ST-SCSR on ST datasets from normal brain tissues generated by 10 $\times $ Visium platform. (A) Visualization of manually annotation of slice 151675 of DLPFC. (B) Visualization of spatial domains identified by various algorithms. (C) Distributions of accuracy of various algorithms for all 12 DLPFC slices in terms of ARI (left) and NMI (right). (D) UMAP visualizations trajectory inference with PAGA for various algorithms. (E) Distributions of features obtained by various algorithms between the ground truth Layer1 and Layer2, where Kolmogorov–Smirnov test is for significance. (F) Heatmaps of correlation matrices obtained by ST-SCSR (left) and Pearson Coefficient of expression profiles (right), respectively.

Fig. 2(A) visualizes the truth ground spatial domains of slice 151675 of DLPFC dataset, where each domain corresponds to different color. By applying these algorithms to slice 151675, ST-SCSR achieves the best performance, showing the proposed algorithm is precise to characterize structure of spatial domains in brain (Fig. 2B, Supplementary Figs S3–S6). In details, ARI of ST-SCSR is 0.660 (NMI=0.712), whereas that is 0.595 (NMI=0.653) and 0.450(NMI=0.542) for STAGATE [25] and SpaGCN [13], respectively. The reason why STAGATE and SpaGCN independently learn the spatial and expression features of spots, ignoring the heterogeneity of them, which dramatically decrease performance of algorithms. In contrast, ARI of MNMST significantly promotes to 0.631 (NMI=0.683) because it transforms spatial coordinates and expression profiles of genes into multi-layer networks, thereby reducing heterogeneity of ST data. Interestingly, ST-SCSR outperforms MNMST in terms of various criteria, demonstrating that the meta-structure of spots can further improve the performance of algorithms for spatial domain identification.

Except for slice 151675, we also apply these algorithms to all slices of DLPFC dataset, and accuracy of various algorithms is shown in Fig. 2(C), where the left panel is for ARI, and right for NMI, respectively. It is easy to conclude that ST-SCSR achieves the best performance, demonstrating that the proposed algorithm is insensitive to measurements and slices of DLPFC dataset. In details, ARI of ST-SCSR is 0.583 $\pm $ 0.093 (median $\pm $ standard deviation), whereas the ARI of the best two baselines MNMST and STAGATE is 0.564 $\pm $ 0.005 and 0.500 $\pm $ 0.057, respectively. These results further prove that the fusion strategy proposed by ST-SCSR is more precise than current methods. NMIs of various algorithms for DLPFC datasets are shown in the right panel of Fig. 2(C), where the tendency is highly consistent with that of ARI. In other words, ST-SCSR also achieves the best performance, followed by MNMST and STAGATE. In details, NMI of ST-SCSR is 0.672 $\pm $ 0.097, while it is 0.638 $\pm $ 0.003 and 0.593 $\pm $ 0.103 for MNMST and STGATE, respectively. These results further demonstrate the superiority of ST-SCSR for identifying spatial domains in brain ST data.

Then, we check quality of features of spots learned by various algorithms by using UMAP, where spatial domains identified by SCANPY and ST-SCSR is visualized with UMAP [38] in Fig. 2(D), where spatial domains identified by SCANPY are mixed, and these identified by ST-SCSR are clearly delineated (Materials and Methods section). These results demonstrate that features learned by ST-SCSR are more discriminative than those obtained others, proving that fusion of heterogeneous information from the meta-structure of spots level is promising. And, we further demonstrate the quality of features learned by various algorithms by showing distribution of features across various spatial domains. Fig. 2(E) describes the distribution density of features learned by SCANPY and ST-SCSR between the ground truth Layer1 and Layer2. Interestingly, distribution of features learned by ST-SCSR between these two layers is significant ($P$ = 0.02, Kolmogorov–Smirnov Test), whereas it is non-significant for features learned by SCANPY ($P$ = 0.7, Kolmogorov–Smirnov Test). These results demonstrate that the proposed algorithm effectively learns discriminative features of spots.

One of the typical difference between ST-SCSR and baselines is that it automatically the shared factor matrix, which can be interpreted as similarity of spatial domains. Thus, we compare the learned correlation matrix of spatial domains and similarity matrix of truth ground spatial domains with Pearson coefficient of expression profiles as shown in Fig. 2(F), where left panel is heatmap of correlation matrix obtained by ST-SCSR, and right for Pearson coefficient. Strictly, Pearson coefficient cannot discriminates these ground truth spatial domains because the minimum value is 0.95, showing that similarity of spatial domains cannot be obtained directly from expression profiles (right panel of Fig. 2F). However, heatmap of correlation matrix learned by ST-SCSR is block diagonal, demonstrating that each domain is highly similar only with the adjacent ones, and these domains far away from each other is dissimilar. For example, correlation between Layer1 and Layer2 is 0.73, whereas it is 0.18 between Layer1 and Layer5. These results demonstrate that ST-SCSR precisely models relations among spatial domains, which cannot be fulfilled with baselines.

We further validate performance of ST-SCSR on more complex mouse brain datasets, where annotations are from the brain anatomical references of Allen Mouse Brain Atlas (Supplementary Fig. S7). Spatial domains identified by various algorithms are visualized in Supplementary Fig. S7. As expected, ST-SCSR can well characterize the structure of tissues and identify some narrow spatial domains. ST-SCSR identified ’cord-like’ structures and ’arrow-like’ shapes in the hippocampus of mice. Specifically, the ’cord-like’ structure corresponds to the pyramidal layer of Ammon’s horn. It mainly contains three spatial domains, CA1, CA2, and CA3 (i.e. CA1sp, CA2sp, and CA3sp). The ’arrow-like’ structure corresponds to the granule cell layer of the dentate gyrus (i.e. DG-sg). Although STAGATE and PROST algorithms also identified the ’rope-like’ structure and the ’hook’ shape. However, after reduce the number of spatial domains to 15, STAGATE identified CA1, CA2, CA3, and DG-sg as one domain, while PROST could not identify this structure. ST-SCSR could identify CA1, CA3, and DG-sg regions. Overall, these results illustrate the ability of ST-SCSR to identify organizational structures and reveal their organization from different data from 10$\times $ Visium. This showing that ST-SCSR is promising for identifying complicated spatial domains in brain.

### ST-SCSR accurately dissects cancer spatial domains from human breast cancer ST dataset

**Figure 3:**
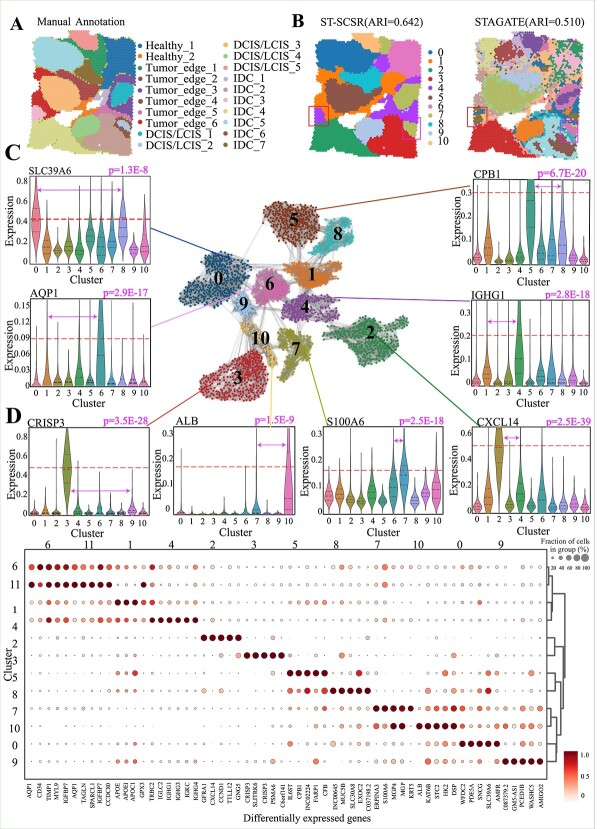
ST-SCSR accurately identifies cancer and non-cancer spatial domains from human breast cancer data. (A) Visualization of human breast cancer annotated by pathologists with IDC, DCIS, LCIS, tumor edge, and healthy region. (B) Visualization of spatial domains identified by various algorithms. (C) Topological structure of spatial domains identified by ST-SCSR in affinity graph, and expression distribution of bio-marker genes for domains. (D) Heatmap of DEGs for various spatial domains, where color is proportional to expression, and sizes of circles is proportional to fraction of cells, respectively.

Previous experiment validate performance of ST-SCSR on the identification of spatial domains from normal brain tissue, and then we testify its performance with the breast cancer dataset to check whether ST-SCSR discovers cancer related spatial domains. Fig. 3(A) is the visualization of H&E breast cancer tissue, consisting of ductal carcinoma in situ/lobular in situ (DCIS/LCIS), healthy region (Healthy), invasive ductal carcinoma (IDC), and tumor surrounding regions (Tumor edge).

Prior to describing performance of various algorithms on the breast cancer dataset, we first determine the number of clusters (spatial domains). Visualization of spatial domains identified by ST-SCSR as the number of clusters increases from 9 to 20 is performed (Supplementary Fig. S8), where increasing the number of clusters from 9 to 20 results in over-segmentation of spatial domains. In other words, some small spatial regions (over-segmented from healthy regions) provide finer-granularity, but fail to provide additional meaningful distinctions, which is also consistent with DeepST [28]. Therefore, we set the number of clusters as 11. Performance of various algorithms for the breast cancer dataset is illustrated in Supplementary Fig. S9(A), where ST-SCSR and MNMST are the two best algorithms, which outperforms other baselines. In details, ARI of MNMST and ST-SCSR is 0.642 and 0.662 respectively, whereas it is 0.572 (BANKSY), 0.510 (STAGATE), 0.594 (DeepST), 0.574 (PROST), 0.517 (SEDR), 0.592 (stLearn), 0.516 (SCANPY), and 0.563 (SpaGCN), respectively. Notice that although ST-SCSR is inferior to MNMST, difference is subtle, while it is much better than other baselines. Moreover, ST-SCSR These results demonstrate that ST-SCSR also precisely identifies spatial domains in breast cancer dataset.

Similar to brain datasets, we also check whether ST-SCSR learns relations of spatial domains (Supplementary Fig. S9B), where all these spatial domains are divided into two groups, i.e. normal (domain 1, 4, 6) and cancer (other domain) group. It demonstrates ST-SCSR also accurately learns correlation of cancer spatial domains, providing clues for exploiting heterogeneity of tissues. Furthermore, we also investigate discriminative of features learned by ST-SCSR by checking its performance on delineating the ground truth Healthy, DCIS/LCIS, and IDC regions (Supplementary Fig. S9C), where distribution of features significantly differs ($P$ = 1.1E-24: Healthy vs IDC, $P$ = 2.0E-15: DCIS/LCIS vs IDC, $P$ = 6.5E-26: DCIS/LCIS vs IDC, Kolmogorov–Smirnov Test), proving that ST-SCSR precisely captures intrinsic structure of complicated cancer spatial domains.

ST-SCSR automatically learns affinity graph of spots by manipulating spatial and expressional features, and performs graph clustering on the affinity graph to obtain cancer related spatial domains. To select the best number of clusters, we visualize spatial domains identified by ST-SCSR by varying the number of clusters from 9 to 20 as shown in Fig. 15, where we find that increasing the number of clusters from 9 to 20 results in over-segmentation of spatial domains. Therefore, 11 clusters reaches a good balance among these critical regions, including Tumor, Tumor Edge, and Healthy regions, without over-complicating the segmentation. Therefore, it is natural to check whether these spatial domains can be reflected by topological structure of affinity graph. Then, we visualize topological structure of these 11 spatial domains identified by ST-SCSR as illustrated in Fig. 3(C), where each domain corresponds a cluster of affinity graph because connectivity within each cluster is strong, and weak across various clusters. These results further prove that spatial domains can be effectively modeled and characterized by topological structure of graphs, which is the reason why network-based model is popular for ST data. To further validate biological meaning of spatial domains, we also check bio-marker genes for each domain, where these genes significantly express in the corresponding region (Student’s $t$-test for significance). Typical bio-marker genes, such as, are highly related to breast cancer. For example, overexpression of SLC39A6 can promote migration and invasion of breast cancer cells due to its regulation of multiple signaling pathways such as EMT (epithelial-mesenchymal transition) and cytoskeletal remodeling via zinc ions [44]. CXCL14 encodes secreted proteins involved in immunoregulatory and inflammatory processes, promoting tumor growth in breast cancer [45]. ALB exists in healthy tissues and encodes albumin. Its main function is to maintain the colloidal osmotic pressure of plasma and transport a variety of small molecules.

To obtain comprehensive understanding of bio-marker genes for spatial domains (Materials and Methods section), we perform differential expression analysis to obtain DEGs. Figure 3(D) visualizes top 5 DEGs for each spatial domain, where the color of circle is proportional to expression, and sizes denote fraction of cells in the group.

### ST-SCSR is applicable to datasets from various platforms

**Figure 4:**
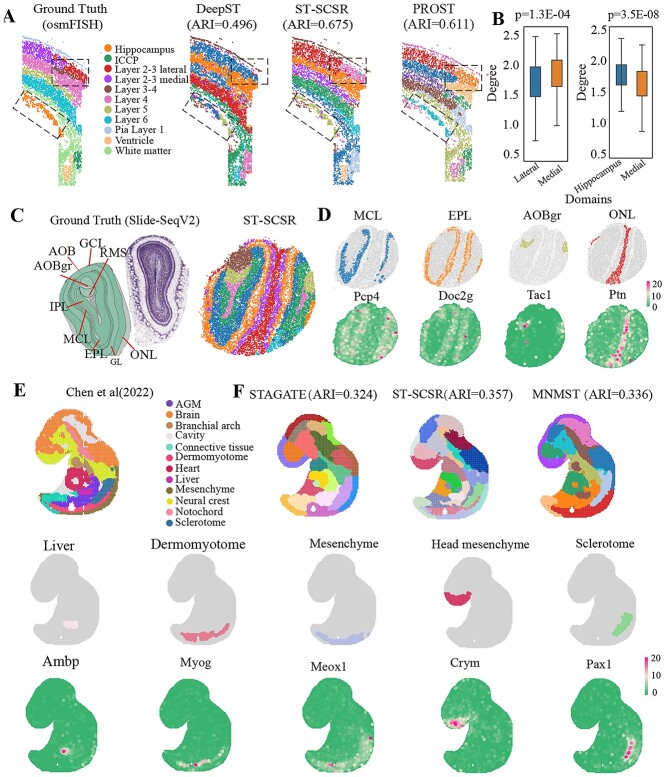
ST-SCSR is applicable to various ST datasets generated by different platforms from different species. (A) Visualization of mouse somatosensory cortex dataset with osmFISH platform, and spatial domains identified by various algorithms. (B) Distribution of degree of spots between the ground truth Lateral and Medial (left), and Hippocampus and Medial (right, student’s *t*-test for significance). (C) Visualization of ST data for mouse olfactory bulb generated by Slide-SeqV2 platform (left), spatial domains identified by ST-SCSR (middle), and expression patterns of bio-marker genes for various domain (right). (D) Visualization of ST dataset for mouse embryos and mouse olfactory generated by Stereo-seq platform (left), and spatial domains identified by various algorithms. (E) Visualization of expression patterns of bio-marker genes for each domain.

Recent advances in ST techniques produce various platforms, and it is natural to testify power of generalization of ST-SCSR to datasets generated with different platforms. First, we apply ST-SCSR to the non-lattice dataset for mouse somatosensory cortex generated by osmFISH platform [46, 47] (Fig. 4A, left panel), where layers are annotated with different colors. Visualization spatial domains identified by various algorithms is shown in Fig. 4(A), where ST-SCSR achieves the best performance. In details, ARI of ST-SCSR is 0.675, whereas it is 0.338 (BANKSY), 0.161 (stLearn), 0.105 (SCANPY), 0.480 (STAGATE), 0.443 (SpaGCN), 0.494 (DeepST), 0.611 (PROST), and 0.619 (MNMST), respectively. ST-SCSR accurately identifies the hippocamus region, while the benchmark algorithm identified the region as two small regions These results demonstrate that ST-SCSR also precisely characterizes structure of spatial domains in osmFISH, demonstrating that the proposed algorithm is insensitive to platforms. Furthermore, we check whether spatial domains can be represented by topological structure of affinity graph learned by ST-SCSR by comparing topological indexes of various domains. Fig. 4(B) shows that degrees of spots between Laterial and Medical regions differ greatly ($P$ = 1.3E-4, Student’s $t$-test), and so does between Hippocampus and Medical regions ($P$ = 3.5E-8, lateral: 1.7 $\pm $ 0.7 vs medial: 1.9 $\pm $ 1.0, Student’s $t$-test). Furthermore, distribution of features learned by various algorithms between the ground truth Laterial and Medial region shows that only ST-SCSR and MNMST significantly discriminate these two domains ($P$ ¡ 0.05, Kolmogorov–Smirnov Test). These results demonstrate that ST-SCSR provides a better way to model ST data, thereby improving the interpretability of spatial domains.

Then, we apply ST-SCSR to ST dataset for mouse olfactory bulb generated by Slide-SeqV2 [48], where annotations are from Allen reference map [49] (left panel of Fig. 4C). ST-SCSR accurately identifies the accessory olfactory bulb and the accessory olfactory granule layer in Fig. 4D). To verify accuracy of spatial domain recognized by ST-SCSR, expression of bio-marker genes corresponding to each spatial domain is visualized, where expression patterns of these bio-marker genes are consistent with structure of domains, including bio-marker gene Doc2g in the outer nuclear layer region, and Pcp4 in the MCL region. These results further prove that ST-SCSR is also applicable for Slide-Seq datasets.

Finally, ST-SCSR is also tested with the dataset generated from the high-resolution spatial transcription platform Stereo-seq [11], which is from mouse embryos with 9.5E (Fig. 4E left panel). STAGATE and MNMST recognizes spatial domains in Stereo-seq dataset, which do not match well with the annotated domains, where some regions are merged (ARI=0.324,0.336). In contrast, ST-SCSR improves ARI to 0.357. Furthermore, boundary of spatial domain is more smooth than those identified by STAGATE. match well with the annotation of mouse embryos. More importantly, spatial domains identified by ST-SCSR are in concordance with the known bio-marker genes as shown in Fig. 4(F). Particularly, the liver region is marked by Ambp, dermomyotome by Myog, mesenchyme by Meox1, Meox2, head mesenchyme by Crym, Sclerotome by Pax1, heart by Myl7, Nppa, and connective tissue by Wnt2, respectively. These results further demonstrate that ST-SCSR is applicable for Stereo-seq dataset, indicating that it is promising for analyzing ST data.

## Discussion

In this study, we propose a novel algorithm (called ST-SCSR) for spatial domain identification from ST datasets, which integrates local information, global information as well as meta-structure of spots. Compared to current algorithms, ST-SCSR presents a new strategy to fuse heterogeneous information, i.e. matrix tri-factorization, where fusion is performed at the meta-structure level, rather than at the spot level. In this case, heterogeneity of ST data dramatically reduces, thereby improving performance of spatial domain identification.

By applying ST-SCSR to various datasets, ST-SCSR is superior to available baselines, demonstrating that the proposed model is promising for analyzing ST datasets. We show ST-SCSR precisely identifies spatial domain from normal brain tissues of human and mouse (Fig. 2). It precisely identifies cancer related spatial domains from human breast cancer dataset, where complicated domains are precisely characterized by topological structure of affinity graph (Fig. 3). Finally, we testify generalization of ST-SCSR for various datasets generated by different platforms, where ST-SCSR still achieves the best performance (Fig. 4). These results demonstrate ST-SCSR not only precisely identifies spatial domain, but also extracts interesting patterns.

Notice that there are many ways to further improve the performance of ST-SCSR. First, ST-SCSR implicitly addresses relation among spatial domains, hampering down-stream analysis. How to explicitly incorporate information of spatial domains into the shared factor matrix is very interesting. Second, ST-SCSR avoids heterogeneity of ST datasets with meta-structure of spots, and how to balance meta-structure of spots and spots themselves is also promising. Third, additional information, such as histological images, is also critical for further enhance performance of ST-SCSR.

**Key Points**

- We present a novel strategy to fuse expression and spatial information at the meta-structure of spots, which is fulfilled with matrix tri-factorization.
- We propose a novel framework to integrate local information, global information, and down-stream tasks for learning compatible features of spots, which improves discriminative of features.
- The experimental results demonstrate that ST-SCSR is more precise to up-to-date baselines for the identification of spatial domains and patterns.

## Supplementary Material

Supplementary_Materials_bbae437

## Data Availability

The code for the ST-SCSR algorithm and a detailed tutorial are available at https://github.com/xkmaxidian/ST-SCSR.
