# Supplementary material for: ST-SCSR: identifying spatial domains in spatial transcriptomics data via structure correlation and self-representation
Source: Brief Bioinform. 2024 Sep 4;25(5):bbae437. doi: 10.1093/bib/bbae437 (PMC11372132; doi:10.1093/bib/bbae437)
Supplement: Supplementary_Materials_bbae437 [file supplementary_materials_bbae437.docx]

Supplementary Materials for

Spatial Domain Identification of Spatial Transcriptomics Data Based on Matrix Factorization and Joint Sparse Representation

# 1 Supplementary materials of ST-SCSR

## 1.1 Mathematical model for ST-SCSR

This section describes the details of the ST-SCSR algorithm. The input of ST-SCSR algorithm mainly includes two parts: gene expression matrix and spatial location information. Firstly, the k-nearest neighbor algorithm is used to construct a spatial neighborhood graph of the cell's spatial position. The weight values of edges in the cell space neighborhood graph correspond to the similarity of the spatial positions of two cells. In order to better explore the correlation between cell types and spatial domains, the gene expression matrix and spatial location were simultaneously subjected to non-negative matrix factorization to obtain a common matrix *G*, which describes the correlation between spatial domains and cell types. The objective function can be expressed as:

| \| $\min\parallel X-B^{[e]}CF^{[e]}\parallel^{2}+\parallel W-B^{[s]}CF^{[s]}\parallel^{2}+\alpha Tr\left( F^{\left[ e \right]}L_{W}{(F^{[e]})}^{'} \right)$  $+\beta\left( F^{[e]}-F^{[e]}Z\parallel^{2}+\parallel F^{[s]}-F^{[s]}Z\parallel^{2} \right)+\gamma\parallel Z\parallel_{1}$  $s.t.B^{[e]}\geq0,B^{[s]}\geq0,F^{\left[ e \right]}\geq0,F^{[s]}\geq0,C=C^{'},Z=Z^{'}$ \| (S1) \| \| --- \| --- \| |
| --- | --- | --- |

The matrix$C$is obtained in joint learning, which includes both the correlation between cell types and the correlation between domains. Where *k* is the number of spatial domains. The joint self-representation learning of $F^{[e]}$ and $F^{[s]}$ generates a powerful and informative representation $Z$, which can improve the performance of downstream tasks. $Z$ integrates complementary information from $X$ and $W$, thereby improving the model's robustness to data noise and outliers. The objective function of learning the cellular spatial expression network $Z$ is shown in formula 2. $\parallel\parallel_{1}$ is $l_{1}$-norm. In order to project the high-dimensional gene expression matrix into a low dimensional space while preserving local similarity between cells, a local preservation projection was also performed on the gene expression matrix $X$. Where $L_{w}$ =$D$ - $W$ is the Laplace matrix, ($D$ is the degree diagonal matrix of $G$). $Tr(A)$ represents the trace of matrix *A*. Where $\alpha$, $\beta$ and $\gamma$ is a parameter for tuning. The objective function is optimized using the alternating direction multiplier method (ADMM), and the update rules for the objective function are detailed in the supplementary materials.

## 1.2 Optimization of ST-SCSR model

Equation (9) was solved using the alternating direction method of multipliers. By introducing auxiliary variables $J$ , the solution to equation (9) is equivalent to the following problem:

| $\min\parallel X-B^{[e]}CF^{[e]}\parallel^{2}+\parallel W-B^{[s]}CF^{[s]}\parallel^{2}+\alpha Tr(F^{[e]}L_{W}{(F^{[e]})}^{'})$  $+\beta(F^{[e]}-F^{[e]}Z\parallel^{2}+\parallel F^{[s]}-F^{[s]}Z\parallel^{2})+\gamma\parallel J\parallel_{1}$  $s.t.B^{[e]}\geq0,B^{[s]}\geq0,F^{[e]}\geq0,F^{[s]} \geq0,Z=J$ | (S2) |
| --- | --- |

The augmented Lagrange function of Eq. (16) is formulated as

| $\begin{aligned} \mathcal{\mathcal{L}(}B^{[e]},C,F^{[e]},B^{[s]},F^{[s]},Z,J)=&\parallel X-B^{[e]}CF^{[e]}\parallel^{2}+\parallel W-B^{[s]}CF^{[s]}\parallel^{2} \\ &+\alpha\mathrm{tr}(F^{[e]}L_{G}{(F^{[e]})}^{'})+\beta(\parallel F^{[e]}-F^{[e]}Z\parallel^{2}+\parallel F_{s}-F_{s}Z\parallel^{2}) \\ &+\gamma\parallel J\parallel_{1}+<T,Z-J>+\mu\parallel Z-J\parallel^{2} \end{aligned}$ | (S3) |
| --- | --- |

Where $\mu$ represents a positive penalty scalar. T is a Lagrangian multiplier, $<>$represents the inner product of a matrix, and ST-SCSR updates a variable by controlling other variables until the objective function reaches convergence. We optimize the objective function by alternately updating $B^{[e]}, C, F^{[e]}, B^{[s]}, F^{[s]}$, and $Z$. The problem in the equation can be solved using the multiplier alternating method.

| $\left\{ \begin{aligned} &&&&B^{[e]}\leftarrow\arg\min_{B^{[e]}} \parallel X-B^{[e]}CF^{[e]}\parallel^{2} \\ F^{[e]}\leftarrow\arg\min_{F^{[e]}} \parallel X-B^{[e]}CF^{[e]}\parallel^{2}+\alpha Tr\left( F^{[e]}L_{W}{(F^{[e]})}^{'} \right)+\beta\parallel F^{[e]}-F^{[e]}Z\parallel^{2} \\ C\leftarrow\arg\min_{C} \left\Vert X-B^{[e]}CF^{[e]} \right\Vert^{2}+\left\Vert W-B^{[s]}CF^{[s]} \right\Vert^{2} \\ B^{[s]}\leftarrow\arg\min_{B^{[s]}} \parallel W-B^{[s]}CF^{[s]}\parallel^{2} \\ F^{[s]}\leftarrow\arg\min_{F^{[s]}} \parallel W-B^{[s]}CF^{[s]}\parallel^{2}+\beta\parallel F^{[s]}-F^{[s]}Z\parallel^{2} \\ Z\leftarrow\arg\min_{Z}\beta\left( \parallel F^{[e]}-F^{[e]}Z\parallel^{2}+\parallel F^{[s]}-F^{[s]}Z\parallel^{2} \right)+\gamma\parallel Z-J+\frac{T}{\mu}\parallel^{2} \\ J\leftarrow\arg\min_{J} \left\vert J \right\vert_{*}+{\mu\left\vert Z-J+\frac{T}{\mu} \right\vert}^{2} \\ T\leftarrow T+\mu(Z-I) \end{aligned} \right.$ | (S4) |
| --- | --- |

By fixing other variables to optimize $B^{[e]}$, and setting the partial derivative $\frac{\partial L}{\partial B^{[e]}}$ of the above objective function to 0, the update formula for $B^{[e]}$ is as follows

| $B^{[e]}=B^{[e]}\odot\frac{X{(F^{[e]})}^{'}C^{'}}{B^{[e]}CF^{[e]}{(F^{[e]})}^{'}C^{'}}$ | (S5) |
| --- | --- |

let the partial derivative of $\frac{\partial L}{\partial F^{[e]}}$ be 0, and the update formula for the subproblem $F^{[e]}$ is as follows

| $F^{[e]}=F^{[e]}\odot\frac{{C^{'}B^{[e]}}^{'}F^{[e]}X+\alpha F^{[e]}W+\beta\left( F^{[e]}Z+F^{[e]}Z^{'} \right)}{C^{'}B^{[e]}'B^{[e]}CF^{[e]}+\alpha F^{[e]}D\beta\left( F^{[e]}+F^{[e]}ZZ^{'} \right)}$ | (S6) |
| --- | --- |

let the partial derivative of $\frac{\partial L}{\partial B^{[s]}}$be 0, and the update formula for the subproblem $B^{[s]}$ is as follows

| $B^{[s]}=B^{[s]}\odot\frac{C{(F^{[s]})}^{'}C^{'}}{B^{[s]}CF^{[s]}{(F^{[s]})}^{'}C^{'}}$ | (S7) |
| --- | --- |

let the partial derivative of $\frac{\partial L}{\partial F^{[s]}}$ be 0, and the update formula for the subproblem $F^{[s]}$ is as follows

| $F^{[s]}=F^{[s]}\odot\frac{C^{'}{B^{[s]}}^{'}W+\beta\left( F^{[s]}Z+F^{[s]}Z^{'} \right)}{C^{'}{B^{[s]}}^{'}B^{[s]}CF^{[s]}+\beta\left( {F^{[s]}+F}^{[s]}ZZ^{'} \right)}$ | (S8) |
| --- | --- |

let the partial derivative of $\frac{\partial L}{\partial C}$be 0, and the update formula for the subproblem $C$ is as follows

| $C=C\odot\frac{{B^{[e]}}^{'}X{(F^{[e]})}^{'}\alpha{B^{[s]}}^{'}W{(F^{[s]})}^{'}}{{B^{[e]}}^{'}B^{[e]}CF^{[e]}{(F^{[e]})}^{'}+\alpha{B^{[s]}}^{'}B^{[s]}CF^{[s]}{(F^{[s]})}^{'}}$ | (S9) |
| --- | --- |

let the partial derivative of $\frac{\partial L}{\partial Z}$be 0, and the update formula for the subproblem $Z$ is as follows

| $Z=Z\odot\frac{\beta\left( {B^{[e]}}^{'}{F^{[s]}}^{'}+{B^{[s]}}^{'}{(F^{[s]})}^{'} \right)+\mu J-T}{\beta\left( {(F^{[e]})}^{'}F^{[e]}Z+{(F^{[s]})}^{'}F^{[s]}Z \right)+\mu Z}$ | (S10) |  |
| --- | --- | --- |

The update formula for the subproblem $J$ is as follows

| $J\leftarrow\underset{J}{\mathrm{argmin}}\frac{\gamma}{\mu}\left\Vert J \right\Vert_{*}+\left\Vert J-\left( Z+\frac{T}{\mu} \right) \right\Vert_{2}^{2}$ | (S11) |
| --- | --- |

## 1.3 Identification of spatial domains

After obtaining the matrix $Z$, the affinity graph is constructed as $(|Z\left| + \right|Z|^{'})/2$ since $Z$ is unnecessarily symmetric. The Leiden algorithm is deployed on the affinity graph to obtain spatial domains. The procedure of ST-SCSR is described in Algorithm 1.

| **Algorithm 1** The ST-SCSR Algorithm |
| --- |
| **Require:** Gene expression $X$ and spatial information $W;$ $\alpha,\beta,\gamma:$ Regularization parameters;  **Ensure:**$\{{\mathcal{\mathcal{M}}}_{i}{\}}_{i=1}^{\epsilon}:Clusters$  1: Spatial Position Information Distance Matrix Based on KNN Construction,  2: Enhancing gene expression using spatial location information;  **Part I: Initialization**  3: Initialize $B^{[e]},F^{[e]},C,B^{[s]},F^{[s]},Z,J;$  **Part II: Optimization**  4: Update $B^{[e]}$ according to Eq. (S5),  5: Update $F^{[e]}$ according to Eq. (S6)  6: Update $B^{[s]}$ according to Eq. (S7),  7: Update $F^{[s]}$ according to Eq. (S8);  8: Update $C$ according to Eq. (S9);  9: Update $Z$ according to Eq. (S10);  10: Update $J$ according to Eq. (S11);  11: Go to step 4 until convergence;  12: let $Z=(\vert Z\left\vert+ \right\vert Z\vert^{'})/2$  13: Identify spatial domains with Leiden algorithm based on $Z$;  14: return $\{{{\mathcal{\mathcal{M}}}_{i}{\}}_{i}}^{\epsilon}=1$ |

# 2. Supplementary Figures

| 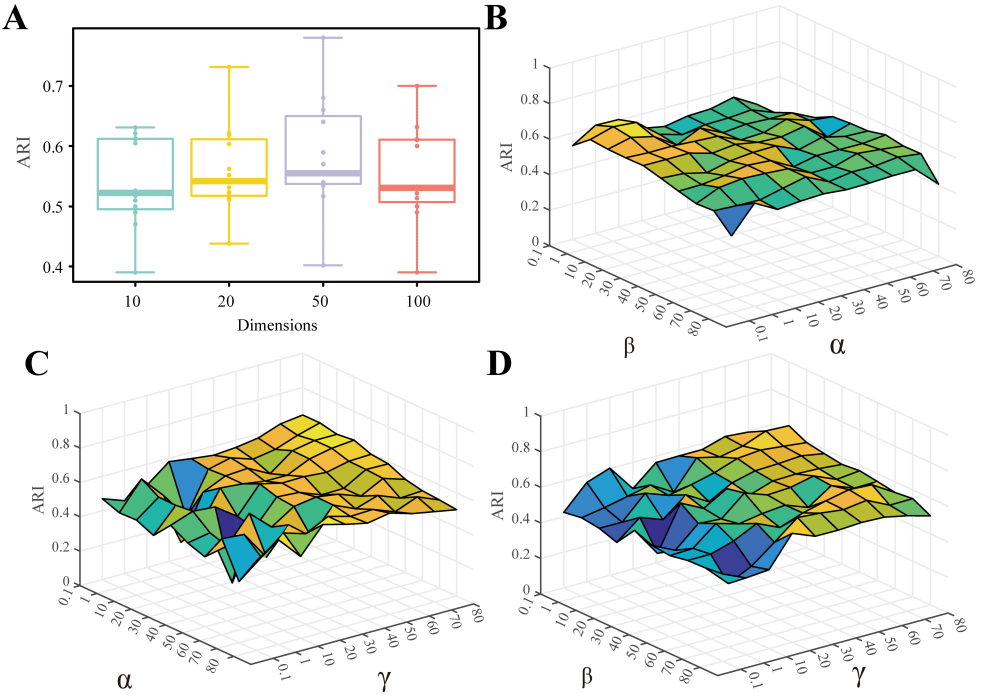  **Fig. S1** Parameter effects of ST-SCSR with DLPFC dataset: (A) ARI vs the number of dimensions, (B) ARI vs parameter (α, β), (C) ARI vs parameter (α, γ), and (D) ARI vs parameter (β, γ).  **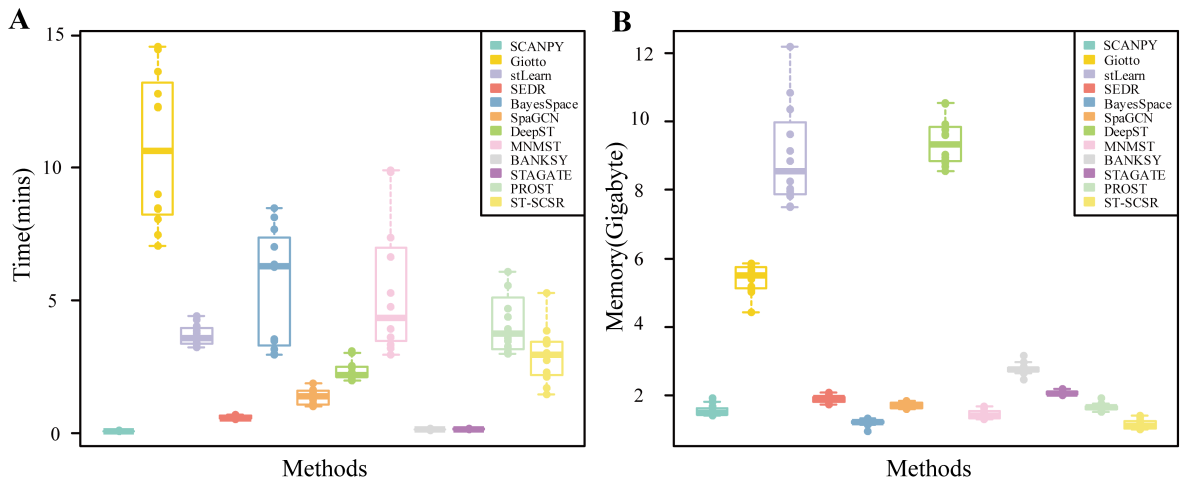**  **Fig. S2** Running time and space of algorithms for different spatial transcriptomics data, where missing bars represents out of memory. (A) Distributions of running time (minutes) of algorithms on the DLPFC data. (A) (B) Distributions of running space (Gigabyte) of algorithms on the DLPFC data.  **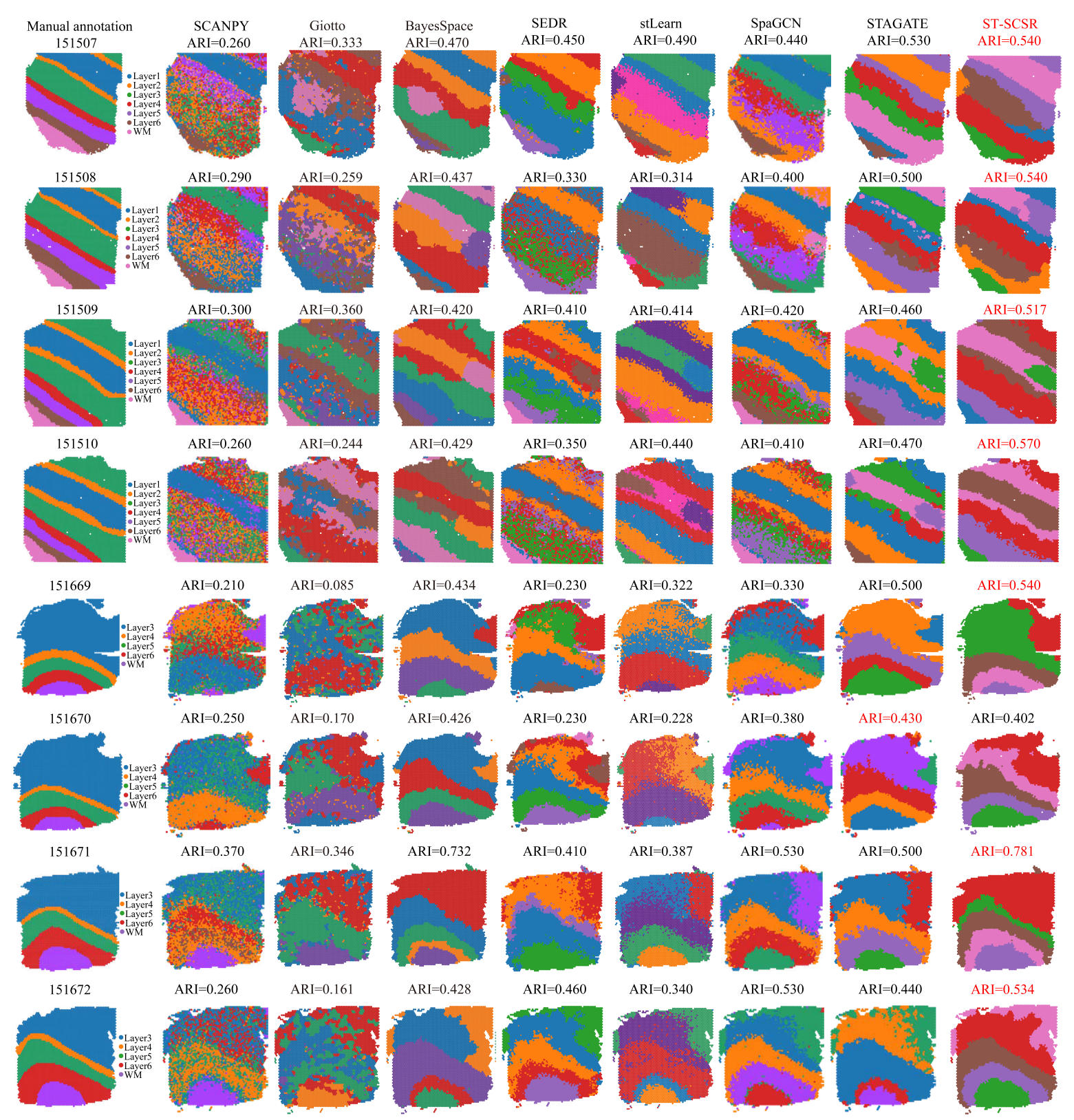Fig. S3** Performance of various algorithms for spatial domain identification on Annotated dorsolateral prefrontal cortex (DLPFC, http://spatial.libd.org/spatialLIBD) data (151507, 151508, 151509, 151510, 151669, 151670, 151671, 151672), where ground truth spots are mapped on their spatial location, divided into various cortical layers (L1-L6 or L3-L6) and white matter (WM) layer, and each column corresponds to performance of an algorithm for various slices in terms of ARI.  **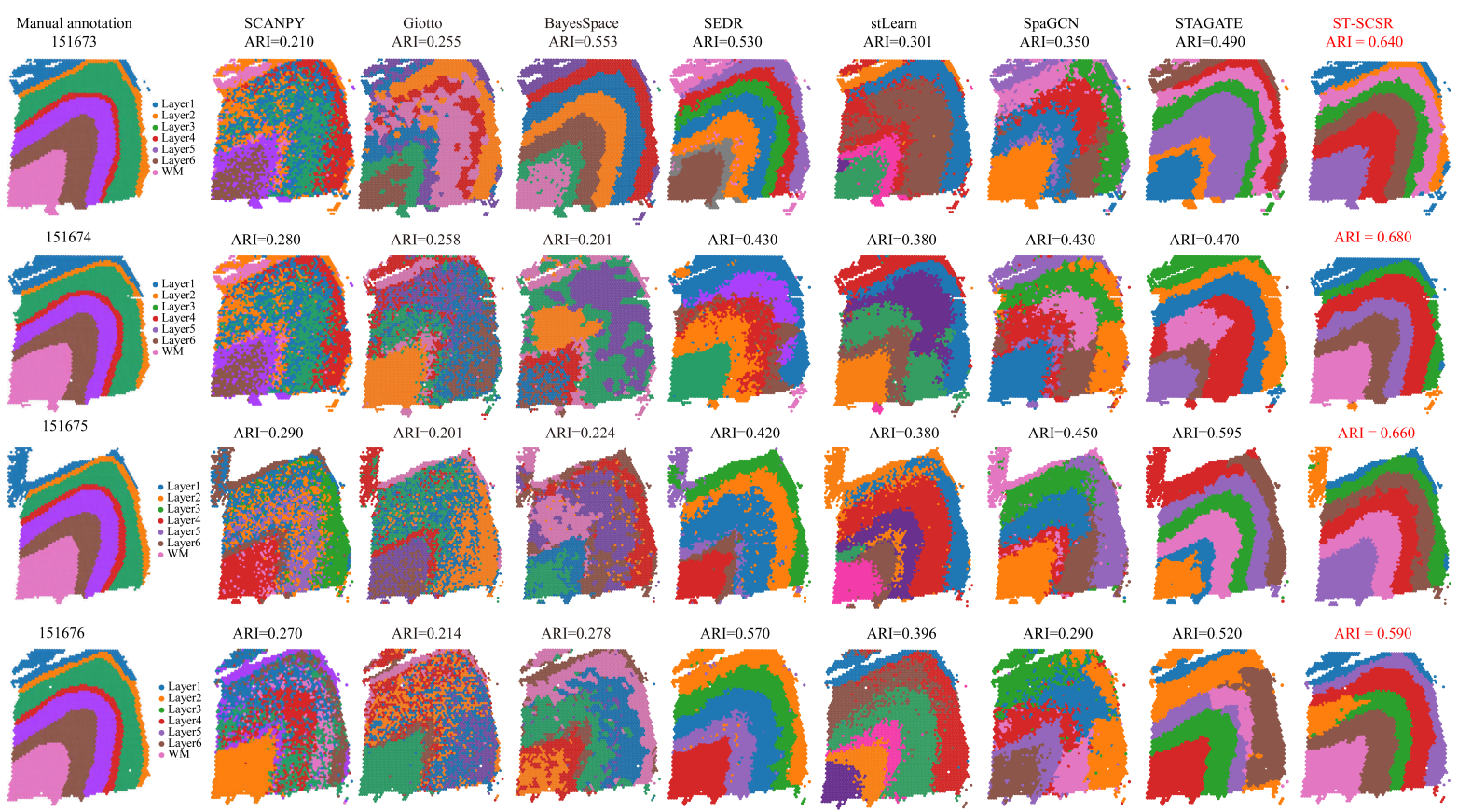 Fig. S4** Performance of various algorithms for spatial domain identification on Annotated dorsolateral prefrontal cortex (DLPFC, http://spatial.libd.org/spatialLIBD) data (151673, 151674, 151675, 151676), where ground truth spots are mapped on their spatial location, divided into various cortical layers (L1-L6) and white matter (WM) layer, and each column corresponds to performance of an algorithm for various slices in terms of ARI.  **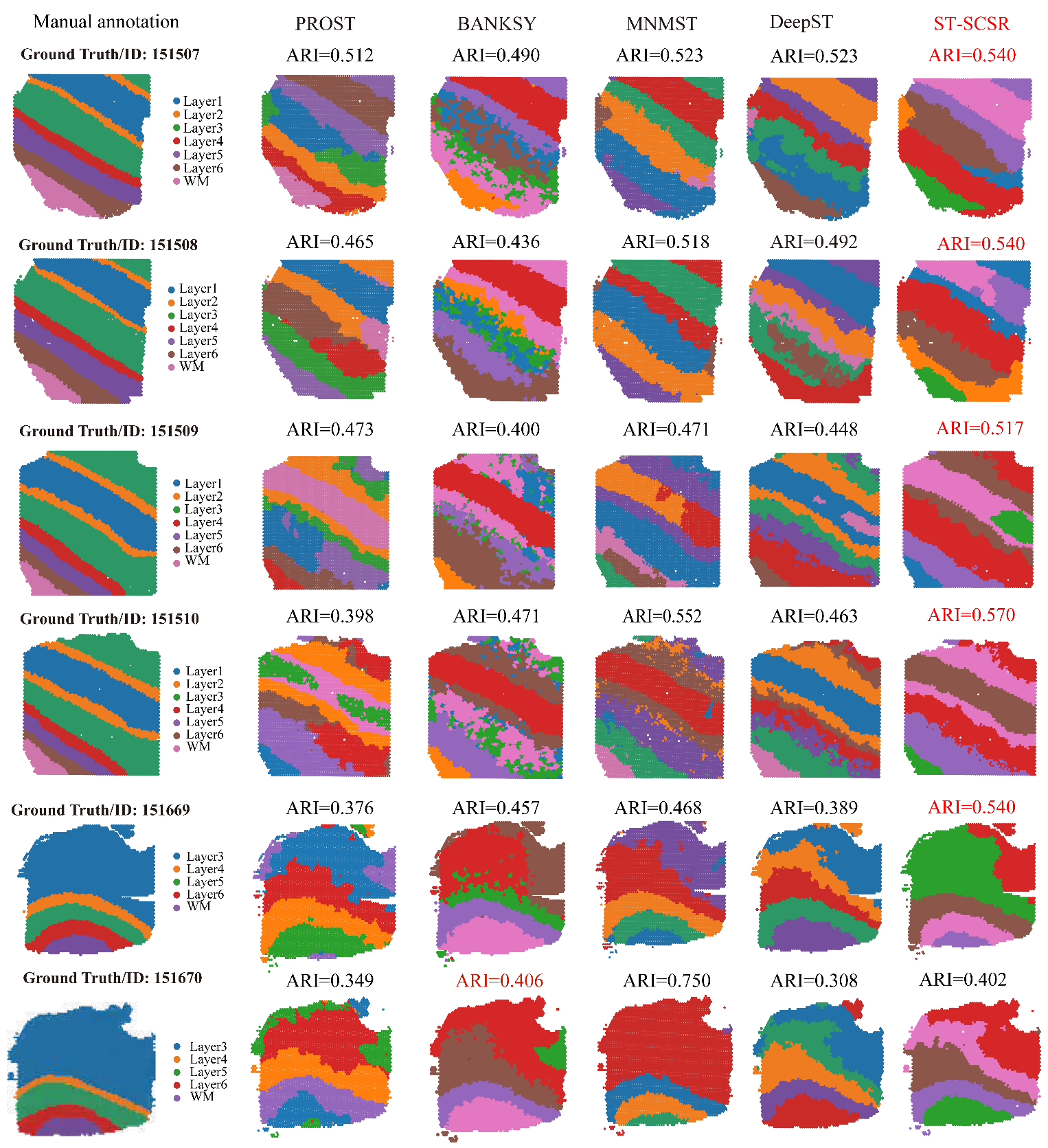 Fig. S5** Performance of various algorithms for spatial domain identification on Annotated dorsolateral prefrontal cortex (DLPFC, http://spatial.libd.org/spatialLIBD) data (151507, 151508, 151509, 151510, 151669, 151670), where ground truth spots are mapped on their spatial location, divided into various cortical layers (L1-L6 or L3-L6) and white matter (WM) layer, and each column corresponds to performance of an algorithm for various slices in terms of ARI.  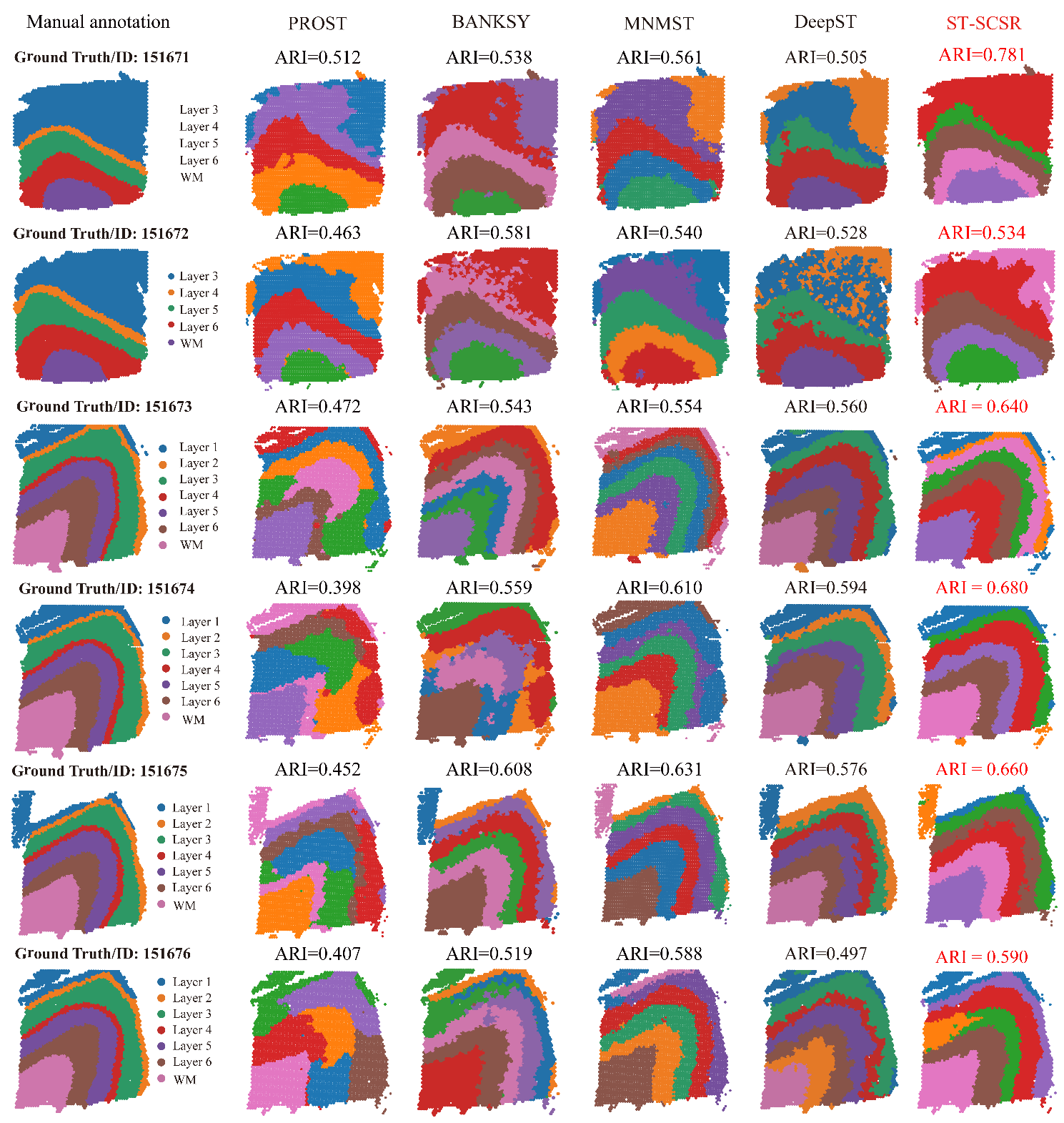 **Fig. S6** Performance of various algorithms for spatial domain identification on Annotated dorsolateral prefrontal cortex (DLPFC, http://spatial.libd.org/spatialLIBD) data (151673, 151674, 151675, 151676, 151671, 151672), where ground truth spots are mapped on their spatial location, divided into various cortical layers (L1-L6) and white matter (WM) layer, and each column corresponds to performance of an algorithm for various slices in terms of ARI.  **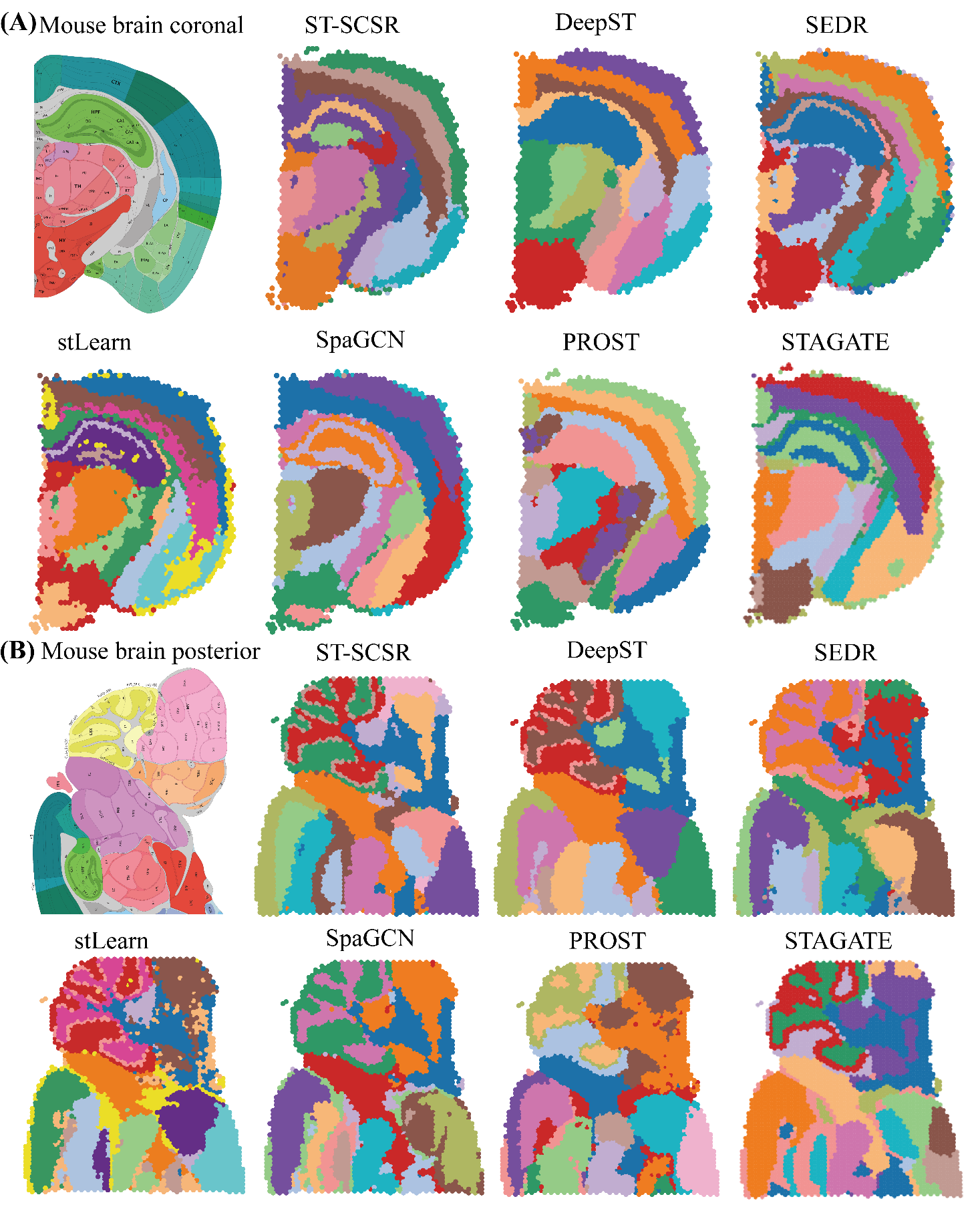**  **Fig. S7** Spatial domain identification for mouse brain tissue. (A) and (B) Spatial domains identified by ST-SCSR, DeepST, SEDR, stLearn, SpaGCN, PROST and STAGATE in mouse brain coronal and posterior, respectively.  **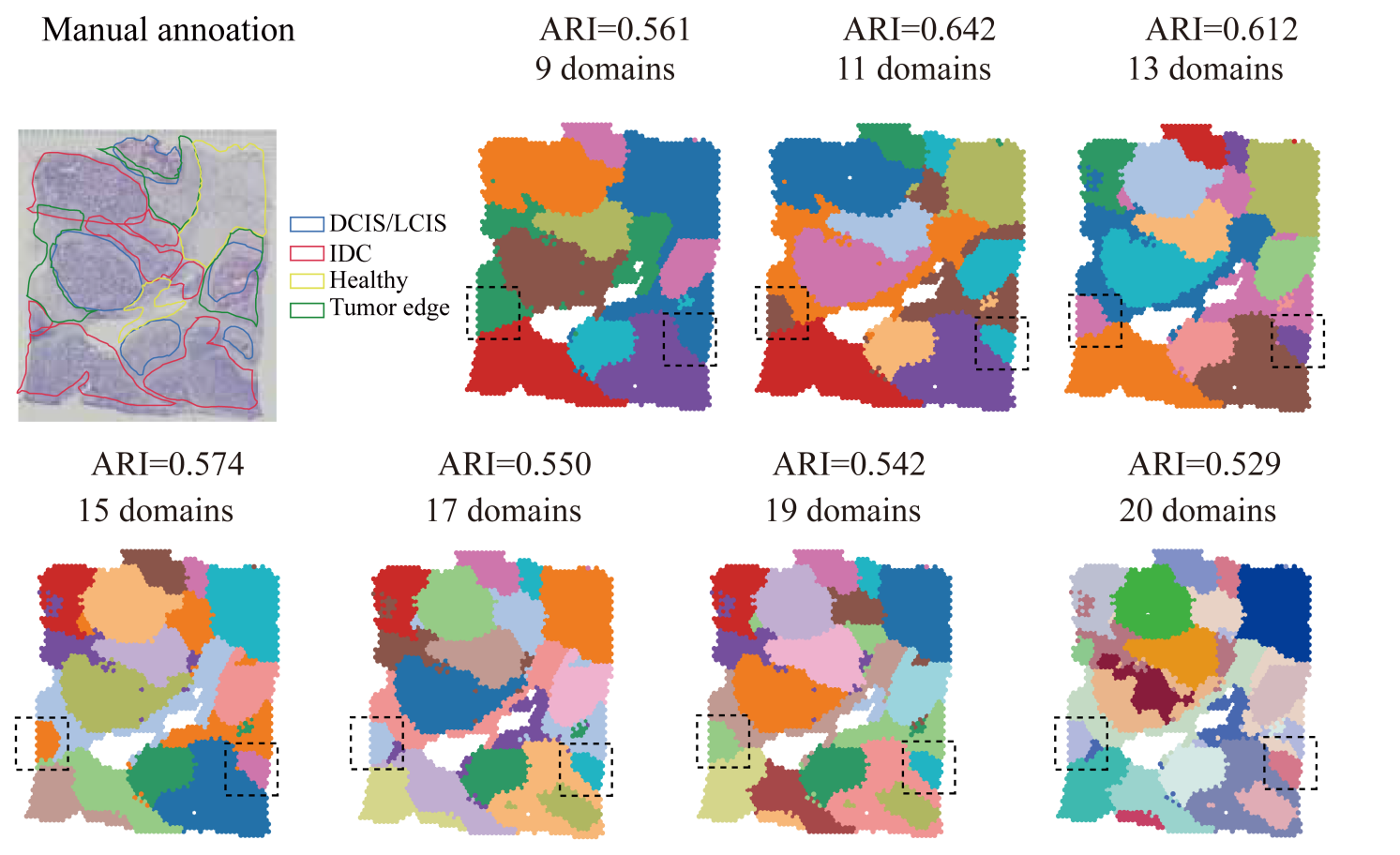**  **Fig. S8** Visualization of spatial domains identified by ST-SCSR by varying the number of clusters from  9 to 20, where dashed squares denote these over-segmented regions.  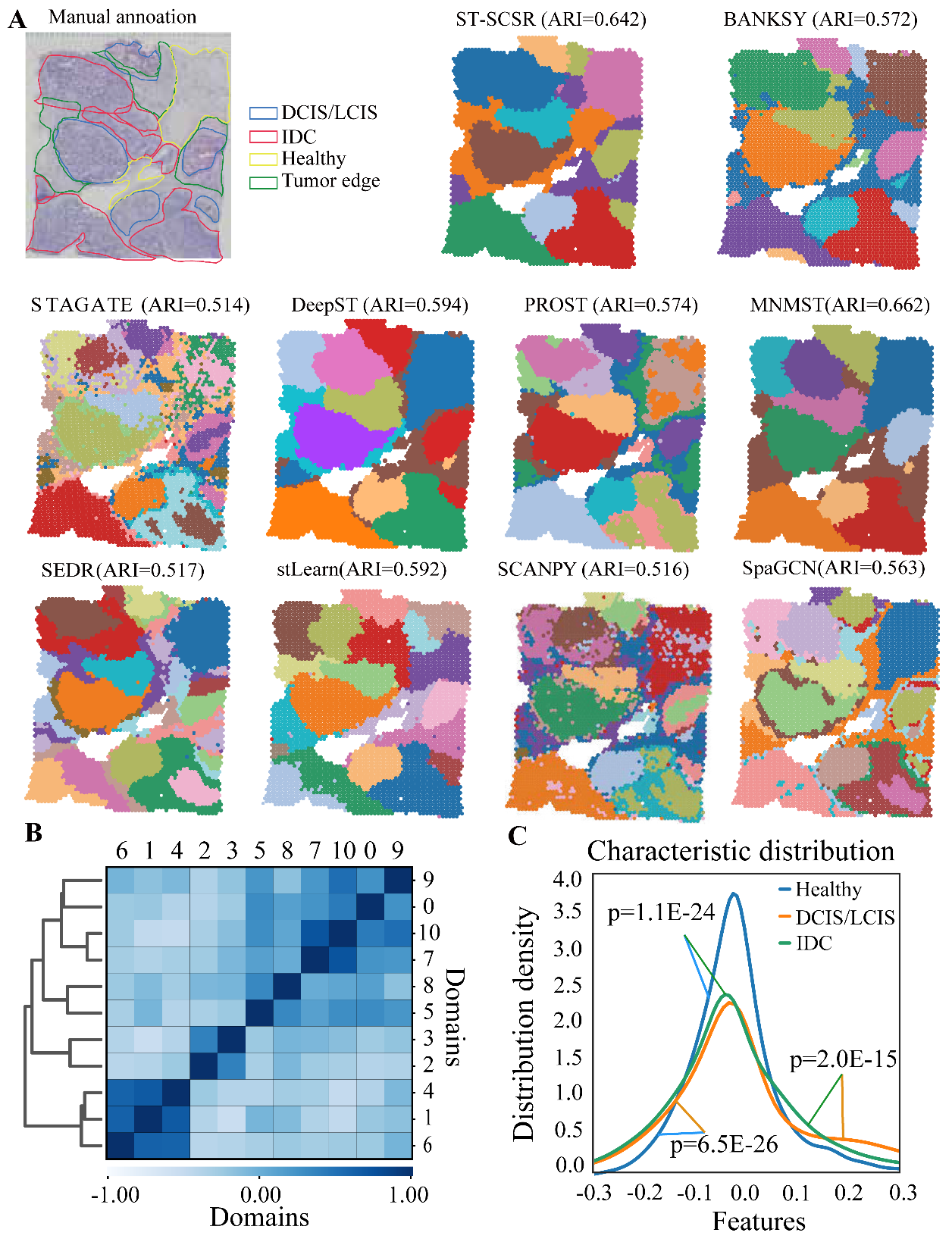  **Fig. S9** Performance of various algorithms for spatial domain identification on the breast cancer dataset. (**A**) Visium SRT data of breast cancer annotated by pathologists consists of IDC (invasive ductal carcinoma), DCIS (ductal carcinoma in situ), LCIS (lobular carcinoma in situ), tumor edge, and healthy region. Visualization of spatial domains identified by state-of-the-art methods, including BANKSY, STAGATE, DeepST, PROST, MNMST, SEDR, stLearn, SCANPY, and SpaGCN respectively. (**B**) Heat map of the correlation matrix in the Breast Cancer spatial domain. (**C**)Distribution density estimation among DCIS/LCIS, Healthy and IDC with features of cells learned by various algorithms.  **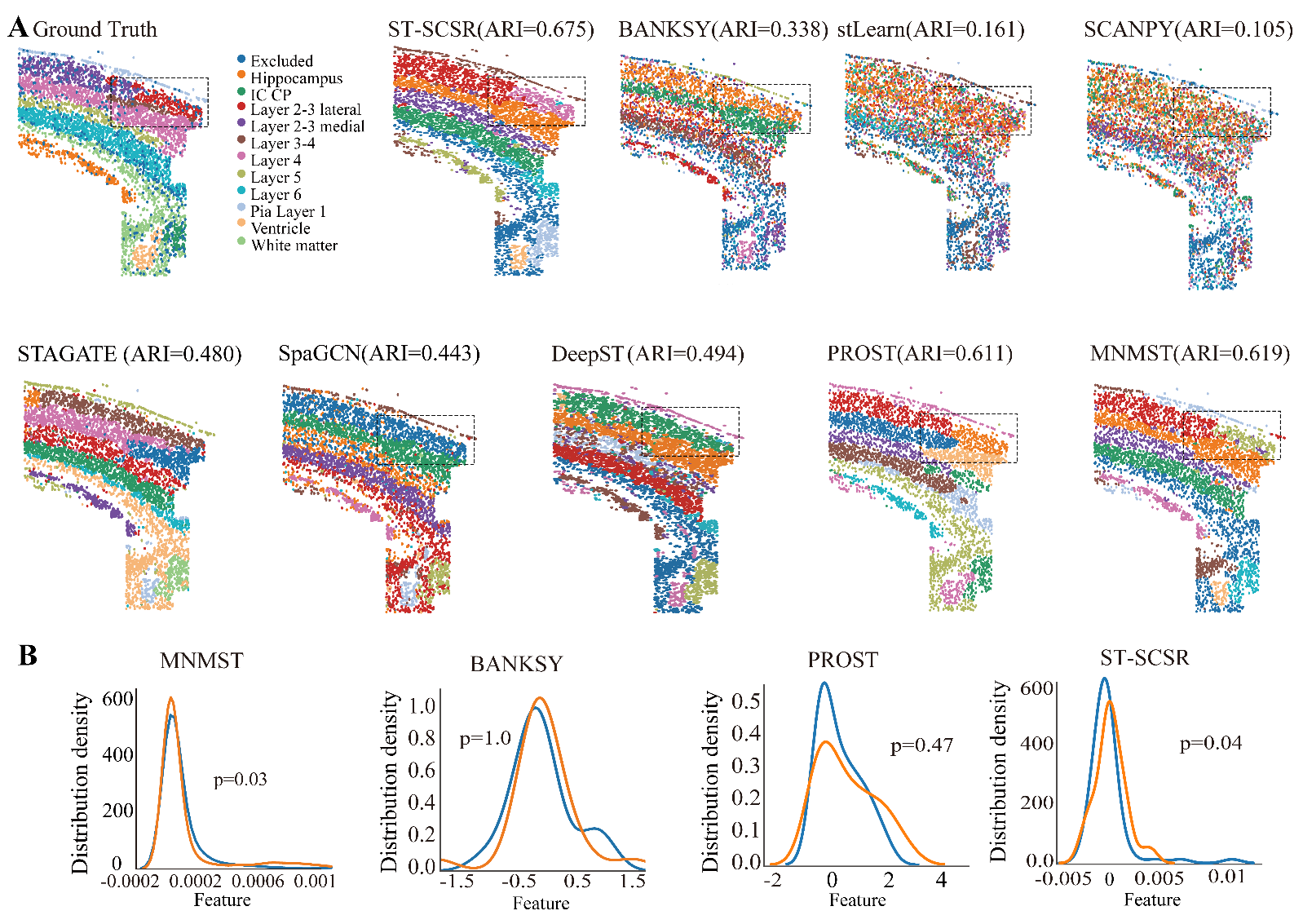Fig. S10** Performance of various algorithms for spatial domain identification on the osmFISH dataset. Ground truth of regional annotation of osmFISH SRT data (left), and spatial domains identified by various algorithms. Distribution density estimation between Lateral and Medial with features of cells learned by various algorithms.  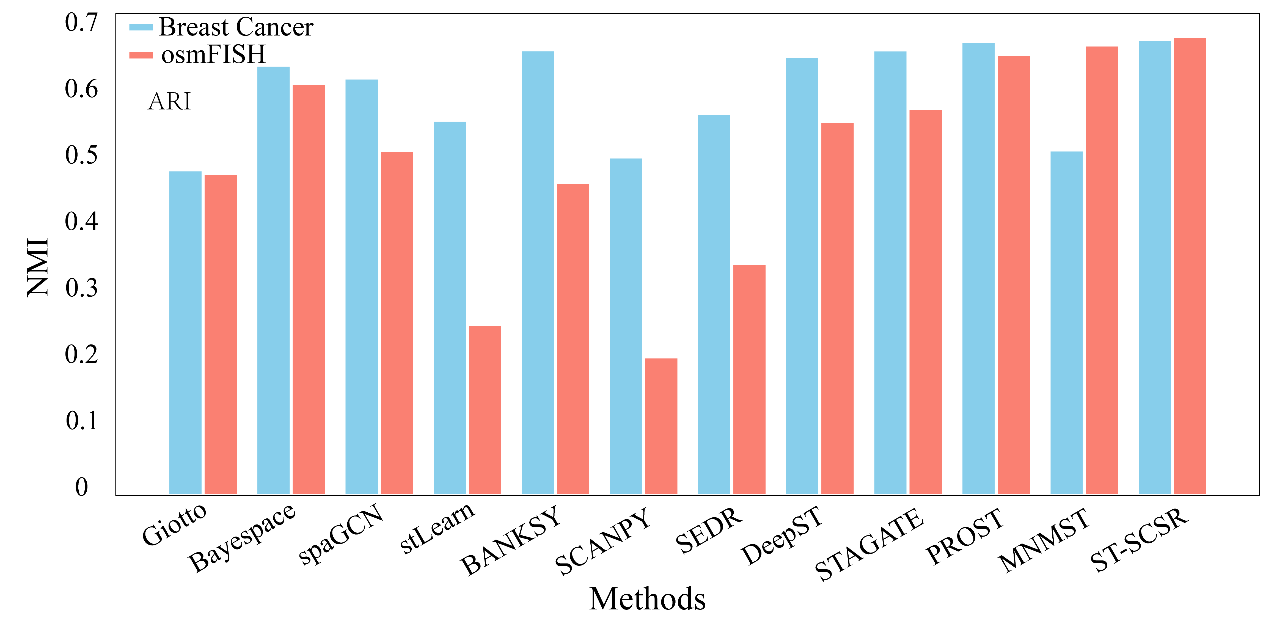 |  |
| --- | --- |

**Fig. S11** Comparison of NMI metrics of various algorithms on the Breast Cancer and osmFISH datasets.
